# A Rare Case of Vanishing White Matter Disease

**DOI:** 10.7759/cureus.67050

**Published:** 2024-08-17

**Authors:** Mrinali Thakur, Vineeta Pande, Shailaja V Mane

**Affiliations:** 1 Pediatrics, Dr. D. Y. Patil Medical College, Hospital and Research Center, Dr. D. Y. Patil Vidyapeeth (Deemed to Be University), Pune, IND

**Keywords:** rare, sudden onset, leukodystrophy, cach, vwmd

## Abstract

Vanishing white matter disease (VWMD), also known as childhood ataxia with central hypoventilation, is a rare leukodystrophy that is inherited in an autosomal recessive manner. It is triggered by either traumatic brain injury or a febrile episode.

The patient was a three-year-old male child who presented with complaints of fever and diarrhea for three days, along with a paucity of movements of both upper and lower limbs, with decreased tone and diminished reflexes. Previously the child had normal developmental milestones. MRI done showed T2 hyperintensities involving bilateral peri-ventricular white matter, deep white matter, and bilateral sub-cortical U-fibres in bilateral fronto-parietal region and bilateral cerebellar hemispheres. The bilateral external capsule and posterior limb of the internal capsule were also involved. All these findings were likely suggestive of leukodystrophy. Whole exome sequencing was done and a homozygous mutation of the eIF2B5 was noted, which confirmed the diagnosis of VWMD.

The physician must keep in mind this diagnosis in cases of sudden motor abnormalities following any event and proceed for early management such as controlling febrile episodes with liberal use of antibiotics and antipyretics, along with prevention of traumatic brain injury or any stressful event. There is no definitive treatment. Management of these patients includes symptomatic and supportive care. Patients with this disease (VMND) have a poor quality of life as the disease progresses and eventually, death occurs.

## Introduction

Vanishing white matter disease (VWMD), also known as childhood ataxia with central hypoventilation, is a rare form of leukodystrophy inherited in an autosomal recessive pattern. It is mainly caused by a mutation in any of the five genes that encode the subunits of the eukaryotic translation initiation factor (eIF2B) [1]. It can be triggered by a traumatic brain injury or a febrile episode. The age of onset ranges from infancy to adulthood. It is reported to have an incidence of 1.2-3.01 per one lakh population per year [2]. VWMD can present as a congenital or early-to-late childhood-onset form. There are even fewer cases reported in India [3-5]. It has been noted to have triggering factors such as traumatic brain injury, febrile episodes, vaccinations, and stressful and frightening conditions. Recent analysis of cerebrospinal fluid in cases of VWMD has shown a consistent elevation of glycine concentration and a ratio of cerebrospinal fluid to plasma glycine concentrations [6], and asialotransferrin levels can be used as biomarkers in cerebrospinal fluid [7,8]. The patient in this case is a three-year-old male child who presented with complaints of fever and diarrhea for three days, along with paucity of movements of both upper and lower limbs, decreased tone, and diminished reflexes.

## Case presentation

The patient was a three-year-old male child, who presented with complaints of fever and diarrhea for three days, along with a paucity of movements of both upper and lower limbs. The child was born out of a second-degree consanguineous marriage with no significant antenatal history or birth history. The child had been immunized till 18 months of age according to the Indian Academy of Pediatrics (IAP) immunization guidelines. The child had attained all milestones till the age of three years in all four domains. On general examination, the child was afebrile and vitally stable. Anthropometric measurements of the child were within normal limits. On neurological examination, the child had an intact sensorium with a Glasgow Coma Scale (GCS) score of 14/15, pupils were reactive to light but decreased tone and diminished reflexes were noted in all four limbs. Power was grade 3 in all 4 limbs. The cranial nerve examination was normal. Other systemic examinations were within normal limits. Diarrhea was treated with antibiotics and laboratory investigations showed the following: whole blood report was suggestive of mild leucopenia along with a normal CRP and no other markers of fever/sepsis were positive. Raised liver enzymes were noted. Dengue and malaria were negative. This is shown in Table 1.

**Table 1:** Hematological investigations on admission

Parameters	Results	Normal range
Hemoglobin	11.3 gm/dl	11-14.5 gm/dl
Total leucocytes count	3400/µL	4000-12000/µL
Platelets	253000/µL	150000-410000/µL
Neutrophils	54%	-
Lymphocytes	40%	-
Monocytes	6%	-
C-reactive protein	3.2 mg/Lt	<3 mg/Lt
Aspartate aminotransferase	353 U/Lt	8-60 U/Lt
Alanine aminotransferase	186 U/Lt	7-55 U/Lt
Ammonia	35 /µL	2-120 /µL
Lactate	3.6 mg/dl	3.6-18 mg/dl
Malaria	Plasmodium vivax: negative; Plasmodium falciparum: negative	-
Dengue	IgM: negative; IgG: negative; NS1: negative	-

MRI brain was done, because of the acute onset of symptoms, with the central nervous system involved. It revealed a reduction in the volume of white matter in bilateral centrum semiovale and in the deep periventricular location with associated altered signal intensity appearing hyperintense on T2-weighted fluid-attenuated inversion recovery (T2 W/FLAIR) images, which was suggestive of leukodystrophy. Differential diagnosis included all leukodystrophies such as Alexander disease, VWMD, Canavan disease, Krabbe disease, and metachromatic leukodystrophy.

Whole exome sequencing was done for a confirmatory diagnosis which showed a homozygous variant of EIF2B5 on exon 7, known to cause leukoencephalopathy with VWMD (Table 2).

**Table 2:** Whole Exome Sequencing

Gene and transcript	Exon/intron number	Variant nomenclature	Zygosity	Classification	Disease	Inheritance
EIF2B5	Exon 7	c.1135A>T p.lle379Phe	Homozygous	Uncertain significance	Leuko-encephalopathy with vanishing white matter	Autosomal recessive

A nerve conduction test was done for all four limbs which turned out to be normal.

The child was managed conservatively as there is no definitive treatment available and was discharged on supplements and physiotherapy exercises. Parents were counseled about the condition and its genetic implications. The importance of control of febrile episodes and the use of antipyretics to prevent progression was explained.

After eight months of diagnosis, the child presented to the hospital with an episode of febrile illness lasting three days, following which the child had further deterioration of the sensorium and shallow breathing. On examination, the child had a GCS of 7/15 with decreased breathing efforts. Vitally, the child had a high-grade fever with bradypnea. On neurological examination, bilateral pupils were reactive to light, and the cranial nerve examination was normal. Upper and lower limbs were hypertonic, and spasticity was noted along with exaggerated reflexes. Other systematic examinations were not significant. The child was intubated and mechanically ventilated, started on antibiotics to treat the infection, and started on antipyretics to control the fever. During the course of admission, the child had an episode of generalized tonic-clonic seizure, for which an antiepileptic was given to the child.

**Figure 1:**
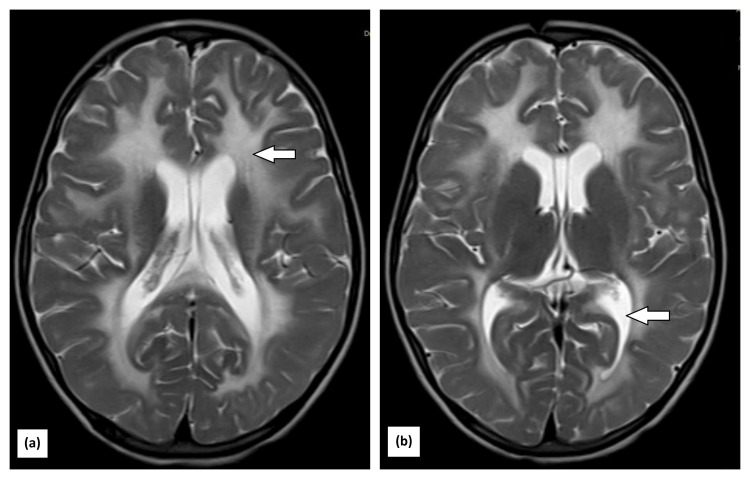
MRI showing (a) multiple ill-defined confluent T2 hyperintensities involving bilateral peri-ventricular white matter, deep white matter, and the bilateral fronto-parietal region. Tigroid appearance noted due to sparing of periventricular white matter; the bilateral external capsule and posterior limb of the internal capsule are involved; and subtle diffusion restriction in the splenium of the corpus callosum and along the lateral margins of white matter hyperintensity in both cerebral hemispheres; (b) bilateral cerebellar hemispheres involving white matter. All these findings support the diagnosis of vanishing white matter disease

A repeat MRI was done that was suggestive of multiple Ill-defined confluent T2 hyperintensities involving bilateral peri-ventricular white matter, deep white matter, and bilateral sub-cortical U-fibres in the bilateral fronto-parietal region and bilateral cerebellar hemispheres involving white matter and bilateral brachium pontis. The tigroid appearance was noted due to sparing of periventricular white matter, bilateral external capsule and posterior limb of internal capsule are involved, and subtle diffusion restriction in the splenium of corpus callosum and along lateral margins of white matter hyperintensity in both cerebral hemispheres (Figure 1).

The presence of fever with acute neurological symptoms and specific neurological findings point toward VWMD.

The child was on mechanical ventilation for five days. He was weaned of ventilation and managed with supportive care and supplements. The child was discharged on day 20 of hospitalization.

## Discussion

The incidence of VWMD ranges from 1.2 to 3.01 per 100,000 people per year [2]. The disease is known to affect children and adults. This fatal disease has been associated with various comorbidities. The range of presentation of the disease includes 1) prenatal/congenital form, 2) subacute infantile form where age is <1 year, 3) early childhood onset form with age between 1 and <4 years, 4) a juvenile-onset form with age between >4 and 18 years, and 5) an adult-onset form where age is ≥18 years. Severe encephalopathy has been noted in the congenital/prenatal form. Patients who present with later-onset forms have an initial normal motor and intellectual development, followed by a sudden neurologic deterioration that has a chronically progressive or subacute course. Motor deterioration is more prominent in the early childhood onset form of cognitive decline, and personality changes are dominant in the adult onset form. The rapid deterioration in the clinical condition is noted during febrile illnesses, following head trauma or major surgical procedures, or by acute and extreme fright [9]. The case presented had similar findings, where initial deterioration started following a febrile episode.

A diagnostic criterion has been proposed where four important features have been noticed: first, a normal or near-normal initial psychomotor development; second, the onset of neurologic deterioration following any event such as traumatic brain injury or febrile episodes that occur in childhood; third, neurologic signs include cerebellar ataxia, spasticity, epilepsy (not always), optic atrophy (not always), and motor functions disproportionately affected; and fourth, an MRI showing bilateral and symmetric cerebral hemispheric white matter signal intensity similar to cerebrospinal fluid. All four criteria should be identified for diagnosis [10]. Findings in the patient were crucial in ruling out other potential diagnoses and confirming VWMD.

The histology of VWMD reveals several key features: rarefaction of deep white matter, microcystic changes in periventricular white matter, spongiform changes in arcuate fibers and the corpus callosum, and the absence of neuronal loss. These pathological changes are characterized by astrocytic dysfunction [11].

There is no definitive cure for VWMD. Only comprehensive medical care helps the child have a better life in terms of quality, which includes avoiding unnecessary suffering and complications. With each febrile episode or traumatic episode, there is further injury to the brain, and mortality is seen within months to years. Hence, it is important to use antipyretics liberally. Targeted therapy has been researched, which targets eIF2B enzymes, and guanabenz has shown promising results that help in motor performance and brain pathology in animal studies [12,13]. Genetic counseling should be provided to the parents. Ongoing studies that analyze cerebrospinal fluid in cases of VWMD have shown a consistent elevation of glycine concentration with an elevated ratio of cerebrospinal fluid to plasma glycine concentrations [6] and biomarkers such as a decreased level of asialotransferrin levels in cerebrospinal fluid [7,8].

Outcome and follow-up

The disease usually starts at a median age of three to four years; 60% of the cases are symptomatic [14]. Motor symptoms are more common in childhood-onset forms, while cognitive problems are more common in adolescent and adult-onset forms. An analysis revealed that mortality is around 13% in cases where the onset is <2 years of age, which is usually after an acute event [14].

## Conclusions

The VWMD, though rare, should be kept in mind while examining cases of sudden motor difficulty following head trauma or other events. The specific MRI characteristics (such as T2 hyperintensities and the involvement of specific brain regions) and the EIF2B5 mutation are hallmark features that confirmed the diagnosis. Avoiding stressful events such as vaccination, absenteeism from contact sports, and liberal use of antipyretics and antibiotics along with anti-seizure medication is advised, which would prevent the progression of the disease. It has no definitive treatment. Only symptomatic and preventive measures can be given; hence, it is essential to diagnose the disease the disease early and counsel parents regarding its progression. Hence, the early recognition of symptoms (such as the acute onset of neurological issues following a febrile episode) helps with better outcomes, even though the disease is progressive and ultimately fatal. Physicians should also help parents improve the quality of life of their children by providing a comprehensive management plan.
